# Antisense Expression of Apple *TFL1*-like Gene (*MdTFL1*) Promotes Early Flowering and Causes Phenotypic Changes in Tobacco

**DOI:** 10.3390/ijms23116006

**Published:** 2022-05-26

**Authors:** Van Giap Do, Youngsuk Lee, Seonae Kim, Hunjoong Kweon, Gyungran Do

**Affiliations:** 1 Apple Research Institute, National Institute of Horticultural and Herbal Science, Rural Development Administration, Gunwi 39000, Korea; seonaekim@korea.kr; 2 Posthavest Technology Division, National Institute of Horticultural and Herbal Science, Rural Development Administration, Wanju-gun 55365, Korea; kweonhj@korea.kr; 3 Planning and Coordination Division, National Institute of Horticultural and Herbal Science, Rural Development Administration, Wanju-gun 55365, Korea; microdo@korea.kr

**Keywords:** *MdTFL1*, early flowering, gene expression, flower morphology, leaf morphology, internode length

## Abstract

Apples (*Malus × domestica* Borkh.) require up to several years for flowering and bearing fruits. The transition from vegetative to reproductive phase is controlled by floral regulators such as *TERMINAL FLOWER 1 (TFL1)* and *FLOWERING LOCUS T (FT)*. *TFL1* mediates the maintenance of vegetative phase, unlike the antagonistic function of *FT* to promote the transition into reproductive phase. In this study, we isolated apple *TFL1*-like gene (*MdTFL1*) to elucidate various phenotypic traits triggered by the antisense expression of *MdTFL1* in tobacco apart from its floral induction function. Early flowering was observed in the tobacco line with *MdTFL1* knockout, indicating the reduced time for transition to vegetative phases. Quantitative reverse-transcription PCR showed upregulation of genes involved in the regulation of floral induction, including *NtAP1, NtSOC1, NFL1,* and *NtFTs*, and downregulation of carotenoid cleavage dioxygenases (*CCDs*) and *CEN*-like genes in transgenic lines. Interestingly, transgenic tobacco expressing antisense *MdTFL1* exhibited distinct morphological changes in lateral shoot outgrowth, internode length, and the development of leaves, flowers, and fruits. The results suggested that using the antisense expression of *MdTFL1* gene is one of the approaches to shorten the vegetable phase and proposed improvement of plant architecture in horticultural crops.

## 1. Introduction

Throughout the life cycle, plants go through distinct developmental transitions from juvenile to mature or vegetative to reproductive stages [1,2]. Among these, flowering is one of the main phenological events directly associated with juvenile-to-mature phase transition. For perennial trees, the juvenile period is substantially long as compared to herbaceous plants. From a horticultural point of view, prolonged juvenility can be a limiting factor in the development of an efficient breeding program for fruit crops considering that floral induction is subsequently triggered after several years of vegetative growth during which trees are unable to bear any floral buds. The transition to reproductive phase is genetically determined and mediated by expression of floral regulators, such as *TERMINAL FLOWER 1* (*TFL1*) and *FLOWERING LOCUS T* (*FT*), controlling meristem identity [3,4,5]. Previous studies have shown that *FT/TFL1* mediates morphological traits such as plant architecture apart from the function of floral induction [6,7].

As the main floral repressor, *TFL1* displays antagonistic activity for floral transition by competitively binding to transcription factor FLOWERING LOCUS D (FD) against FT [3,8] and controlling the expression of downstream flowering integrators *APETALA1* (*AP1*) and *LEAFY* (*LFY*) [9]. *TFL1* has been targeted for early flowering phenotype in loss-of-function studies in many plants such as *Arabidopsis* [10], rice [11], and various horticultural crop species [12,13,14]). In apple (*Malus × domestica* Borkh.), previous studies have reported two *TFL1* homologs, *MdTFL1-1* and *MdTFL1-2*, with the identification and early flowering phenotype induced by the antisense expression of *MdTFL1* [14,15,16].

Although a recent study has suggested a novel role of *TFL1* in plant development associated with its interactive protein networks apart from the original role of floral regulation [17], plant architecture associated and coordinated with the function of TFL1 has not been well studied. We conducted this study to investigate a wide range of phenotypic traits due to antisense expression of *MdTFL1* in transgenic tobacco. Apart from the early flowering induction, we identified distinctive changes in the phenotypes of lateral shoot outgrowth, leaf morphology, and development of flower and fruit in transgenic lines. Microscopic observation and quantitative reverse transcription PCR analyses further revealed differences in plant architecture that were specifically developed in 35S::MdTFL1-antisense-expressing lines. Our results provide a novel insight that leads to a better understanding of the effects of *MdTFL1* on plant development, and they will be useful for future breeding research as well as improvement of plant architecture.

## 2. Results

### 2.1. Construction of Expression Vector

**Figure 1:**
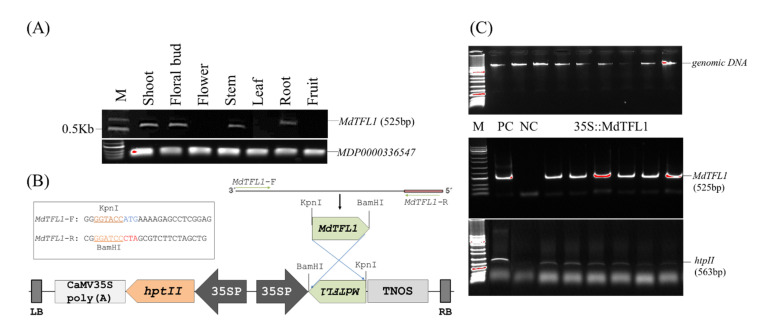
Schematic cassette with a representation of the T-DNA region of expression vector and evaluation of transgenic plants. (**A**) Evaluation of MdTFL1 mRNA transcript via reverse-transcriptase PCR with mRNA transcripts of *MDP0000336547* (SGF29 Tudor-like domain) were used as control. (**B**) Expression vector harboring MdTFL1-antisense located between the cauliflower mosaic virus (CaMV) 35S promoter and the TNOS terminator for nopaline synthase; LB, T-DNA right border; *hptII*, hygromycin phosphotransferase; 35SpolyA, the terminator of 35S gene; LB, T-DNA left border. (**C**) Detection of the target gene (*MdTFL1*) and selection marker hygromycin phosphotransferase (*htpII*) in transgenic lines via genomic DNA PCR analysis. Lane PC, plasmid DNA of 35S::MdTFL1 used as positive controls; lane NC, genomic DNA of WT used as a negative control; lane M, 1 Kb plus DNA ladder marker; lanes 35S::MdTFL1, independent transgenic lines.

**Figure 2:**
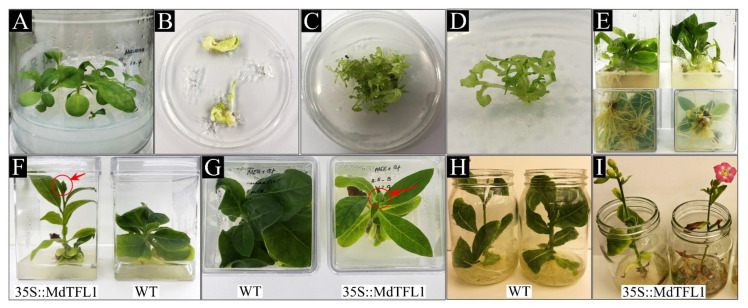
Establishment of transgenic tobacco via in vitro tissue culture. (**A**) Leaf disk explants from a seedling of tobacco *N. tabacum* were cut down and used for plant transformation via the *Agrobacterium*-mediated transformation method. (**B**) Adventitious shoots appeared from induced callus on the selection medium MSSE. (**C**) The adventitious shoots were continuously sub-cultured, and (**D**) putative transgenic shoots were detached from callus and continuously propagated on the MSSE. (**E**) Candidate transgenic plants were transferred to rooting media rooting medium MSR to obtain the whole plantlets with root. (**F**–**I**) After the establishment of plantlets containing root, instead of transferring to the soil, in vitro plantlets were still kept for continuous growth on the culture medium. The transgenic plants showed flowering, whereas no flowering was observed in the WT plants.

To explore the function of *MdTFL1* affecting floral induction along with the overall plant morphology, we constructed a vector expressing *MdTFL1*-antisense. cDNA encoding the *MdTFL1* gene was synthesized from the isolated total RNA of various tissues of ‘Fuji’ apple cultivars using specific primers via RT-PCR. The mRNA expression of *MdTFL1* was variable and tissue specific. Its expression level was strong in the shoot, floral bud, stem, and root but low in the leaves, flower, and fruit (Figure 1A). The cDNA encoding *MdTFL1* gene from the floral bud was isolated, cloned (Figures S1 and S2), and introduced into a plant expression vector under the control of cauliflower mosaic virus 35S promoter (Figure 1B). The expression vector for 35S::MdTFL1 was transformed into tobacco leaf disks. We obtained numerous wild-type (WT) and transgenic lines under the selection process on the selection media containing hygromycin antibiotic via in vitro tissue culture (Figure 2A–E). The leaves of in vitro plants were sampled for genomic DNA isolation. Genomic DNA PCR was performed to confirm the successful insertion of the T-DNA region harboring the selection marker hygromycin phosphotransferase (*htpII*) and target genes (*MdTFL1*) in putative transgenic tobacco plants (Figure 1C). The PCR product showed a positive band with expected sizes for both the selection marker (*htpII*) and target gene (*MdTFL1*). No band of PCR products was obtained from the WT. The results indicated the successful integration of *MdTFL1* gene into the chromosomes of transgenic tobacco plants. The transgenic lines were selected and transferred to the soil for further analysis.

### 2.2. Antisense Expression of MdTFL1 Promoted Early Flowering

**Figure 3:**
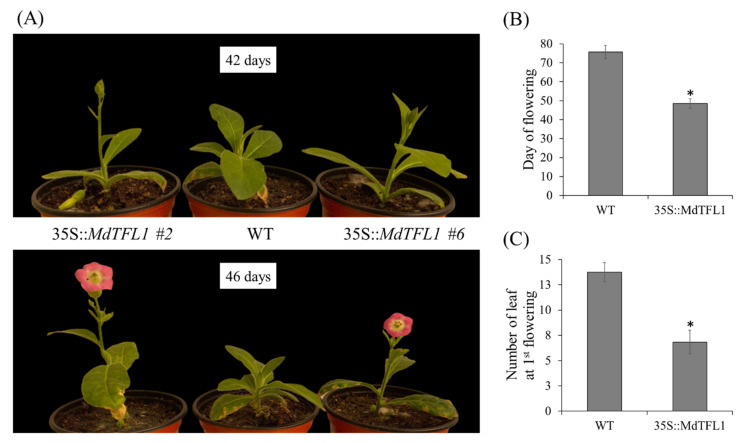
Early flowering of transgenic tobacco expressing *MdTFL1* antisense. (**A**,**B**) The appearance of initial floral buds and flowers from presentative 35S::MdTFL1 transgenic lines and WT at 42 DAT and 46 DAT, respectively. (**C**) Evaluation of flowering time and leaf number at the first flowering of the transgenic lines and WT. Plants were grown in a glasshouse at 25 °C under long-day conditions. The number of leaves was evaluated at the stage when the first flower flowers fully bloomed. Data are presented as mean ± SD (*n* = 20). The asterisks indicate significant differences compared to WT (*p* < 0.05) according to Tukey’s test.

To clarify the function of apple *MdTFL1* gene on regulation of flowering, many transgenic tobaccos were obtained. Transgenic tobaccos with antisense expression of *MdTFL1* flowered much earlier than WT plants (Figure 3). For the two most pronounced transgenic phenotypes (lines #2 and #6), the initial floral bud was observed at 42 days after transferring to the soil (DAT), and flowers fully bloomed with only six to seven leaves at 46 DAT (Figure 3A). In contrast, WT plants were still in the vegetative stage without any flowers, and flowering was delayed for 27 days as compared to transgenic lines. The average flowering times were 48.7 ± 2.3 DAT and 75.8 ± 3.5 DAT for transgenic plants and WT, respectively (Figure 3B). The numbers of leaves for first flowering in both transgenic and WT plants were 6.8 ± 0.8 and 13.8 ± 1.0, respectively (Figure 3C). An early flowering phenotype was observed in vitro in plants (Figure 2F–I). After the establishment of plantlets containing roots, instead of transferring to the soil, some of them were still kept for continuous growth under in vitro conditions on the culture medium. Only the transgenic plants flowered while the WT plant remained at the vegetative stage. To evaluate the genetic stability of transgenic plants, transgenic lines #2 and #6, showing the earliest flowering, were selected, and their seeds were geminated on soil with 30 plants/lines. In homozygous T1 plants, all transgenic lines flowered earlier than WT. The flowering times in 35S::*MdFTL1* transgenic line #2 (#2-T1) and line #6 (#6-T1) were 50.6 ± 0.8 and 50.7 ± 0.5 days after sowing (DAS), respectively, whereas it as 73.6 ± 4.8 in WT (Figure S3). These results indicated that the early flowering phenotype was conserved in transgenic plants, and it was inherited in the next generation.

### 2.3. Antisense Expression of MdTFL1 Induced Lateral Shoot Outgrowth and Affected Plant Architecture

**Figure 4:**
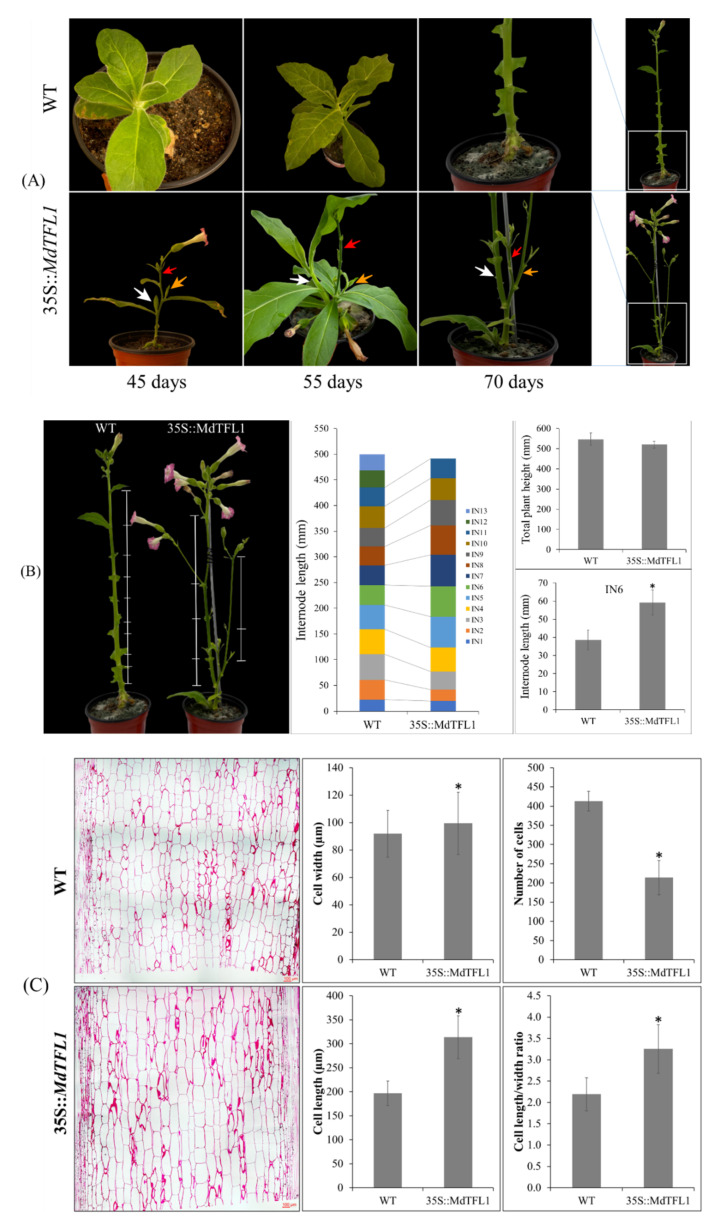
Antisense expression of *MdTFL1*-induced lateral shoot outgrowth and affected plant architecture in tobacco. (**A**) In the transgenic lines, together with the appearance of their first flower, lateral shoots started appearance at 45 DAT and after the abscission of the first flowers, the lateral shoot quickly developed and bore inflorescence flowers. Developing lateral shoots was recorded and marked by difference color arrows. Red marked arrows for the main shoot, while and yellow arrows were marked for lateral shoots. (**B**) Overall height of plant with the difference in elongation of internode leads to changes in the architecture of transgenic plant with more branching and inflorescence flowers. (**C**) Evaluation of cell size from the imaging microscope in vertical section of stem tissue/internode from the transgenic and WT. Data are presented as mean ± SD (*n* = 20). The asterisks indicate significant differences compared to WT (*p* < 0.05) according to Tukey’s test.

Tobacco (*N. tabacum*) has a non-woody stem that develops inflorescence meristem at the terminal end of the main shoot after phase transition to reproductive stage to the end-of-life cycle. We observed flowering of 35S::MdTFL1 transgenic tobacco after development of terminal inflorescence meristem (45 DAT), whose main shoot also produced some axillary buds. After the drop of terminal flowers (55 DAT), the main shoot did not grow up, while axillary buds still rapidly differentiated into lateral shoots. Subsequently, the lateral shoots developed into inflorescence meristem, and both the main shoot and inflorescence meristem flowered, while no lateral shoots were formed in the main shoot of WT plants (Figure 4A), indicating that the antisense expression of *MdTFL1* caused lateral shoot outgrowth in transgenic tobacco plants. Additionally, we observed that as compared to WT, transgenic *Arabidopsis* plants showed phenotypic differences such as outgrowth of lateral shoot, axillary bud, and induction of flowering (Figure S4). These results suggested that *MdTFL1* might play multiple roles and its functions were conserved in many plant species.

In many plant species, internode elongation reflects the plant height, which can be an important trait related to biomass and yield production from the agricultural point of view. In our study, we found that the internode length of transgenic line was significantly longer than WT, although there was no significant difference in the overall plant height between transgenic and WT lines, suggesting that WT plants had a higher number of nodes but their internode length was shorter than that of transgenic plants. The internode length of transgenic plants was significantly longer than WT, with significant differences occurring at fifth to seventh internode positions (IN5-7) (e.g., IN6 in transgenic and WT plants was 59.4 ± 6.8 mm and 38.7 ± 5.4 mm, respectively) (Figure 4B). The elongation of internode in stems is a result of mitotic activity and extension of cells in the elongation zone [18]. To further examine any difference in internode length at the cellular level, we compared the difference of internode elongation in both lines at the cellular level with light microscopy. The cell size of transgenic plants was larger in both dimensions (width and length) than that of WT (Figure 4C). Particularly, the cell length of transgenic plants was 1.6 times longer than that of WT. Interestingly, the ratio of length to width of cells significantly differed between transgenic and WT plants (3.2 and 2.2, respectively); however, the number of cells in WT was 1.9 times higher than that in transgenic plants. This was due to the small cell size of WT plants as compared to transgenic plants.

The *MdTFL1* gene was originally known as a gene involved in delayed flowering with a prolonged vegetative phase. Therefore, suppressing its antisense expression has been implemented as a technique to control early flowering in apples [14] and *Arabidopsis* [15]. In addition to an early flowering phenotype, we found that transgenic tobacco with antisense expression of *MdTFL1* exhibited a wide range of multifactorial phenotypes, suggesting that *MdFTL1* might play a multifaceted role in plant development (Figure 3A, Figure 4A, and Figure S4), similar to that reported in a previous study on transgenic tobacco overexpressing *GhFT1* [19]. In our study, the axillary buds further developed into lateral shoots containing inflorescence flowers; however, this was not observed in transgenic tobacco overexpressing *GhFT1* [19]. Taken together, our results suggested that the antisense expression of MdTFL1 not only caused lateral shoot outgrowth but also altered plant architecture by affecting the internode length through cell elongation.

### 2.4. Antisense Expression of MdTFL1 Influences Leaf Morphology

**Figure 5:**
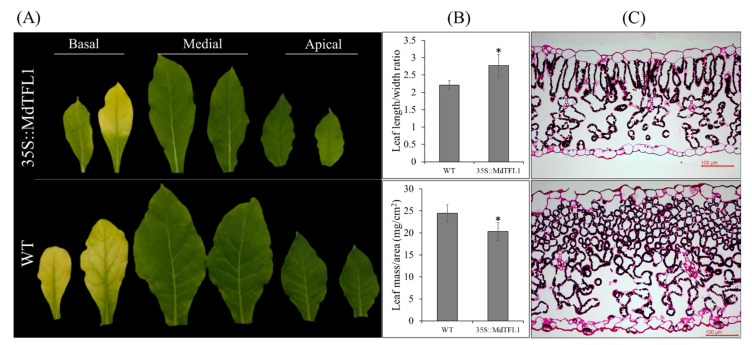
Introducing *MdTFL1* antisense influenced leaf morphology in tobacco. (**A**) Observation of basal/medial/apical leaves was collected from different positions of plants with differences in shape. (**B**) Evaluation of leaf size and LMA. (**C**) The cellular microscope of horizontal sections of leaf tissues. Leaves were sampled for evaluation at the stage of the first flower flowers fully bloomed. Error bars represent standard deviations (*n* = 30). The asterisks indicate significant differences compared to WT (*p* < 0.05) according to Tukey’s test.

Changes in the leaf morphology of 35S::MdTFL1-antisense transgenic plants were observed. In general, the leaf shape and size were different between transgenic and WT lines. The leaf shape in transgenic lines was oblanceolate, while that in WT was rather ovate. Particularly, the sizes of apical, medial, and basal leaves in transgenic lines were smaller than those in WT (Figure 5A). The leaf length-to-width (L/W) ratio was higher in transgenic lines than that in WT (2.7 for transgenic and 2.2 for WT, respectively) (Figure 5B, Table S3), and the leaf mass per area (LMA) of transgenic lines was lower than that of WT (Figure 5B), indicating that antisense-expressing *MdTFL1* affected the development of thin leaves. To further examine any difference at the cellular level that might affect leaf thinness, we evaluated leaf morphology at the cellular level using light microscopy. Indeed, the leaves of transgenic plants were thinner than those of WT with lesser mesophyll tissue layers (Figure 5C). Even though the cell size in WT leaf tissue was small, the mesophyll cell layers were thicker than those of transgenic lines, confirming the significant difference in leaf thickness between the two lines.

### 2.5. Antisense Expression of MdTFL1 Influenced Flower Morphology

**Figure 6:**
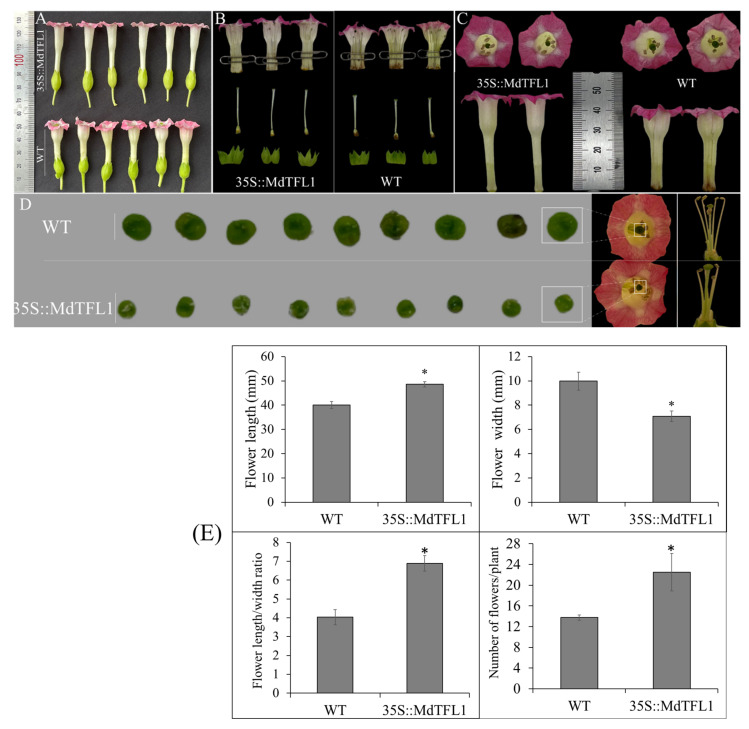
Phenotypic change in the flower of the transgenic tobacco compared to WT including (**A**) overall size, (**B**) anatomy of the floral organs (sepal, petal, carpel ovary), (**C**) size of flower tubo, (**D**) ‘down-pin’ type of filament-style position and closed view of stigma, (**E**) Evaluation of flower morphology. Error bars represent standard deviations (*n* = 30). The asterisks indicate significant differences compared to WT (*p* < 0.05) according to Tukey’s test.

Interestingly, the 35S::MdTFL1-antisense transgenic lines exhibited a distinct development of floral organs. Although there was no difference in the composition of floral organ components (five sepals, five petals, five stamens, and one pistil) (Figure 6A,B), their size and shape differed between transgenic and WT lines. With more detailed anatomy, the sepal of transgenic lines was longer and well-serrated in shape. No significant difference was observed in the ovary size (Figure 6B). However, transgenic lines showed a much smaller stigma than WT (Figure 6C,D). The filament-style position in the flowers of transgenic lines was ‘down-pin’ type (short style-tall filament), whereas the filament-style position in the flowers of WT lines was ‘up-pin’ type (tall style-short filament). The stigma (the terminal of style) was located at a lower position than the anther (the terminal of filament) in transgenic lines, while it was opposite in WT lines. This might affect pollination and fertility ability.

The shape of the floral organ in transgenic lines was longer in length and smaller in width as compared to that of WT (Figure 6C). For a detailed comparison, we measured the size of flower tubo and compared the length to width (L/W) ratio (Figure 6E and Figure S5). The average lengths of tubo in transgenic and WT lines were 48.62 ± 1.08 and 40.03 ± 1.41, respectively, and the average widths of tubo were 7.08 ± 0.43 and 9.98 ± 0.74 for transgenic and WT lines, respectively. Furthermore, the L/W ratio of transgenic lines was 1.72 times higher than that of WT, indicating the potential role of *MdTFL1* in flower development. Additionally, transgenic lines showed a higher number of flowers than WT lines (Figure 6E). The numbers of flowers per plant were 13.8 ± 1.5 and 22.5 ± 3.6 for WT and transgenic lines, respectively. This might also affect the formation of fruits and their yield.

### 2.6. Antisense Expression of MdTFL1 Influenced Set Seed/Fruit in Tobacco

**Figure 7:**
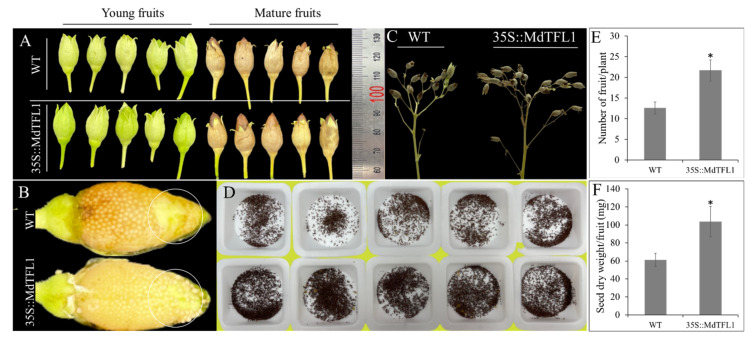
Effect of antisense expression of *MdTFL1* in fruit and seed production. (**A**) Fruits of tobacco at young and mature developmental stages. (**B**) Young, peeled fruits of transgenic lines set more seed than WT at the circled area. (**C**) Fruit-bearing branches. (**D**) Seeds per fruit. (**E**,**F**). Evaluation of set fruit and seed. Error bars represent standard deviations (*n* = 30). The asterisks indicate significant differences compared to WT (*p* < 0.05) according to Tukey’s test.

In addition to the changed phenotypes through antisense expression of *MdTFL1* mentioned above, the differential development of fruit, another reproductive organ related to yield production, was compared in both lines. There was no difference in the fruit shape and size during developmental and mature stages between transgenic and WT lines (Figure 7A). However, the fruit of transgenic lines had more seeds than that of WT (Figure 7B–D). Similar to the comparison of flower development, transgenic lines had a higher number of fruits (21.7 ± 2.6) as compared to WT lines (12.6 ± 1.4) (Figure 7E). Consistently, the weight of fruit seeds in transgenic lines was much higher than that in WT, with increased seed dry weight values of 103.6 ± 16.9 mg and 61.0 ± 7.3 for transgenic and WT lines, respectively (Figure 7F).

### 2.7. Antisense Expression of MdTFL1 Altered the Expression of Genes Involved in Metabolic Pathways for Flowering and Branching

**Figure 8:**
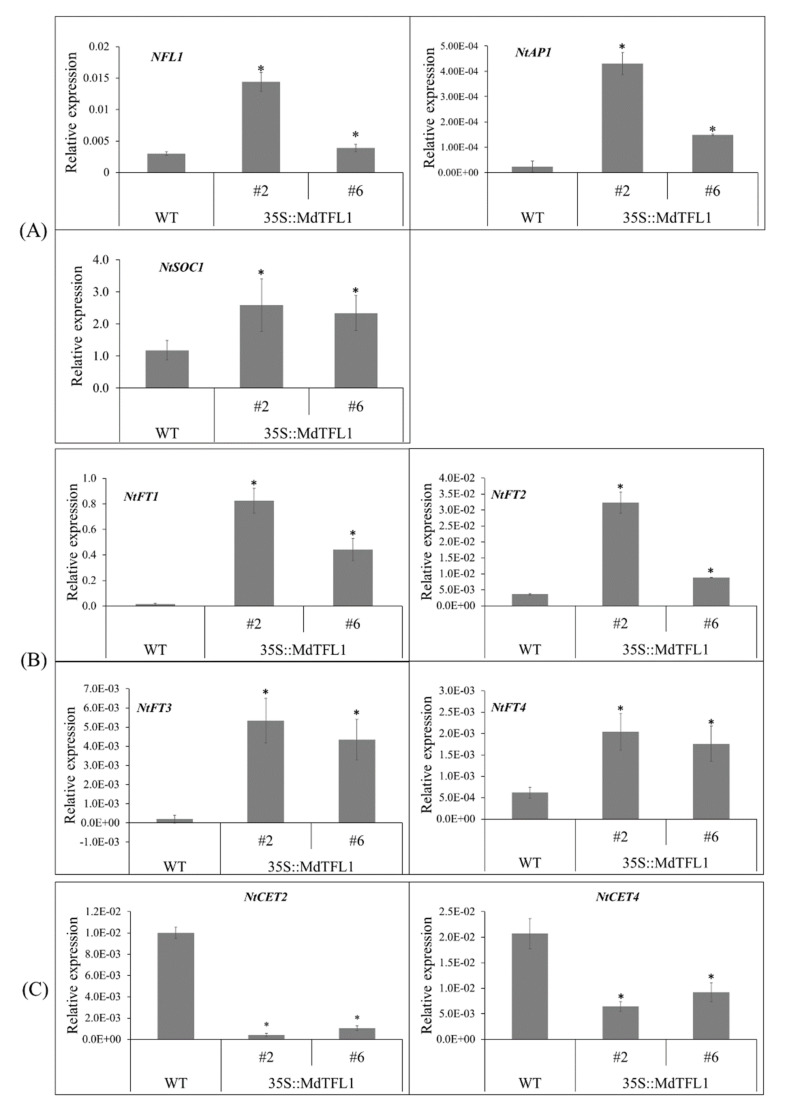

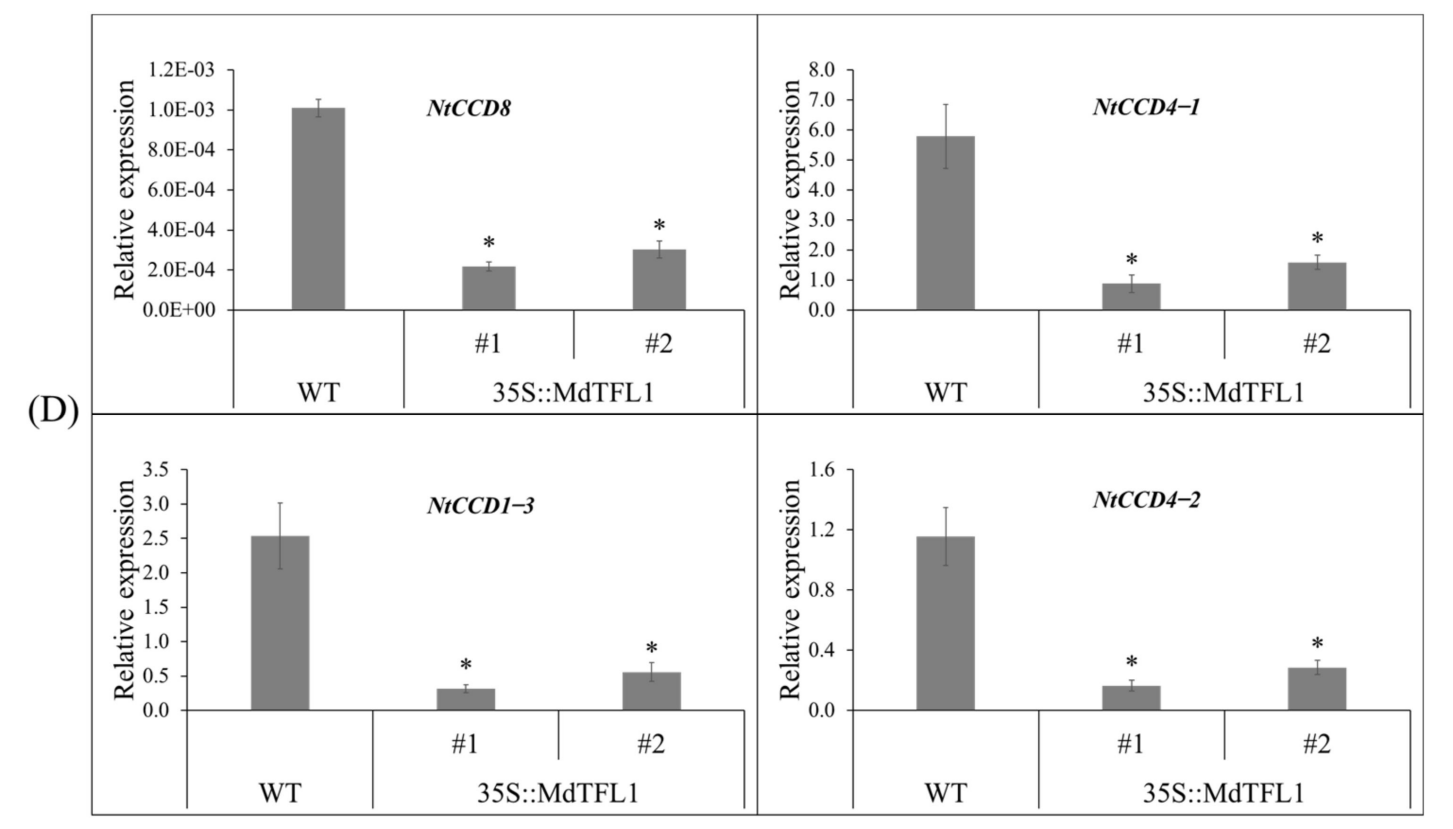
Expression profile of tobacco endogenous-flowering-related gene. The expression level of (**A**) *NFL1*, *NtAP1*, and *NtSOC1*; (**B**) *NtFTs*; (**C**) *CEN*; and (**D**) *CCDs* was normalized to *NtAct*. Data represent the mean ± SD from three biological replicates with seven technical replicates. The asterisks indicate significant differences compared to WT (*p* < 0.05).

Many transduction signals, including floral inducers and inhibitors, are involved in flowering. 35S::*MdTFL1*-antisense triggered the expression level of genes involved in the metabolic pathways for flowering, including floral integrators. The transition from vegetative to reproductive phase regulates floral integrators such as *NFL1, AP1, SOC1*, *FT*, and *CEN*. qRT-PCR analysis showed that the relative expression level of *NFL1, NtAP1*, and *NtSOC1* genes in transgenic lines was higher than that in WT (Figure 8A). Moreover, the expression of *NtFT* genes was upregulated (Figure 8B) and that of *CEN* genes (*NtCET2* and *NtCET4*) was downregulated in transgenic lines (Figure 8C). For branching-related genes, we analyzed *CCD* genes involved in the biosynthesis of strigolactones (SLs), of which phytohormone regulates branching and affects plant architecture [20,21]. The expression levels of *CCD* genes, including *NtCCD8, NtCCD1-3, NtCCD4-1*, and *NtCCD4-2*, were all downregulated in transgenic lines (Figure 8D), consistent with the observation of lateral shoot branching phenotype in *MdTFL1* antisense-expressing lines (described in Section 2.3).

## 3. Discussion

### 3.1. Antisense Expression of MdTFL1 Promoted Early Flowering by Disturbing the Expression Level of Floral Regulator Genes

The flowering time in plants is inherently regulated by the floral regulator *FT/TFL1* family genes. *FT* gene acts as a floral inducer in charge of transition from the vegetative to productive phase, whereas *TFL1* gene functions as a floral inhibitor and maintains the juvenile phase. The *MdTFL1* gene has the same function in apples and *Arabidopsis* [15]. Additionally, antisense expression of *MdTFL1* RNA accelerates flowering in transgenic apple [14]. These observations suggested that antisense expression of *MdTFL1* can promote flowering as a potential floral gene in tobacco. As expected, we found that the antisense expression of *MdTFL1* promoted early flowering in transgenic tobacco. To further investigate the function of *MdTFL1* in regulation of flowering, we evaluated the expression of endogenous floral genes. The expression levels of floral inducers *NFL1, NtAP1*, *NtSOC1,* and *NtFTs* were upregulated in transgenic lines (Figure 8A,B), and the expression level of *CEN* genes was downregulated in transgenic lines (Figure 8C).

*Arabidopsis LEAFY*-like gene (*LFY*) regulates cellular differentiation of shoot apical meristem into floral meristem. *NFL1* is a tobacco *LFY* that shares 73% amino acid sequence and functional identity with *LFY*. The ectopic expression of the *LFY* gene accelerates floral transition, leading to early flowering in tobacco [22]. *CaLFY* overexpression in transgenic tobacco leads to formation of leaf tissues at the floral meristem [23]. In this study, *NFL1* was highly expressed in transgenic lines as compared to that in WT. The *APETALA1 (AP1)* gene, as a floral meristem identity gene, plays a key role in controlling transition from the vegetative to reproductive stage [24]. Overexpression of two poplar *PsnAP1* genes promotes early flowering in transgenic tobacco and *Arabidopsis* [25]. *SOC1* belongs to MADS box genes involved in the regulation of flowering time in *Arabidopsis*. In tobacco, the homologous overexpression of *NtSOC1* promotes flowering [26]. Further, heterologous overexpression of the *FT* gene in transgenic tobacco accelerates flowering with high expression levels of *NFL1, NtAP1,* and *NtSOC1* [19,27]. Moreover, *SOC1* and *FT* are two key floral integrator factors that interact together to activate the downstream floral meristem identity *LFY* and *AP1* genes [28,29].

The *CEN* gene was highly expressed in vegetative axillary meristem, thus delaying flowering. Loss of function of *CEN* genes could promote flowering, whereas their overexpression showed the opposite effects. CEN overexpression in tobacco delays flowering by up to 15 months [30]. In rice, homologous overexpression of *TFL1/CEN* (*RCN1* and *RCN2*) delays heading, promotes branching, and causes panicle with severe defects [11]. In our study, we evaluated the expression of two *CEN* genes, *NtCET2* and *NtCET4*, which shared 97% DNA and 83% amino acid sequence identity to CEN [30]. The expression levels of both *NtCET2* and *NtCET4* genes were downregulated in transgenic lines. Taken together, the antisense expression of apple *MdTFL1* promoted precocious flowering by disturbing the balance of endogenous floral inducers and inhibitors in tobacco.

### 3.2. Antisense Expression of MdTFL1 Gene Not Only Promoted Early Flowering but Also Led to Phenotypic Changes in Transgenic Plants

In this study, the antisense expression of *MdTFL1* not only promoted precocious flowering but also caused a wide range of phenotypic changes in the shoot, leaf, flower, and fruit of transgenic tobacco. First, the transgenic lines exhibited lateral shoot outgrowth and internode length, leading to a change in plant architecture (Figure 4). This was not usually exhibited in WT plants that developed only the main shoot without lateral shoots. In previous studies, the overexpressed *GhFT1* gene causes lateral shoot outgrowth in transgenic tobacco under LD conditions [19], whereas the loss of function of *FT2* exhibited the development of shoot apex of poplar plant [31]. It has been reported that the florigen *FT* gene modulates the outgrowth of lateral shoot and axillary bud in *Arabidopsis* [32,33]. In this study, we found that the expression of *NtFT* genes was upregulated in 35S::MdTFL1 transgenic tobacco (Figure 8B). Therefore, antisense expression of *MdTFL1* gene might modulate lateral shoot outgrowth in transgenic lines in an indirect manner through *NtFT* genes. The results shown in Figure 8D indicated that the introduction of *MdTFL1* gene in transgenic lines downregulated the expression of *CCD* genes because *CCD* genes are required for synthesis of SLs that inhibit axillary bud growth [20]. However, the underlying mechanism of *CCD* regulation by *TFL1* and its genetic interactions remain unclear. Moreover, in our study, repressed *TFL1* gene expression reduced the number of internodes and elongated the length of internodes via elongation of cells (Figure 4B,C). Zhang et al. [11] reported that the overexpression of *TFL1/CEN*-like genes increased the numbers and shortened the length of internodes in transgenic rice. In transgenic tobacco, overexpressing the orchid *FT* gene elongates internodes and promotes axillary buds [27].

Second, we observed that transgenic lines exhibited a broad change in leaf morphology. All obtained transgenic lines exhibited an oblanceolate shape of apical, medial, and basal leaves and ovate shape of WT leaf. Moreover, the leaves of transgenic plants were smaller and thinner with lesser cell layers and lower LMA values than WT plants (Figure 5). Transgenic plants expressing *FT* show reduced leaf sizes in *Arabidopsis* and *Cymbidium* [27,34]. In another study, the leaves of transgenic tobacco overexpressed the *GhTF1* gene with a smaller size but higher LMA value than those of WT. However, the leaves of all 35S::GhFT1 transgenic lines do not exhibit a uniform shape [19].

Third, the flower was structured with the arrangement and integration of several floral organs/units. Surprisingly, many changes occurred in the flower morphology of 35S::MdTFL1-antisense transgenic lines. As a reproductive organ, the flower has been conserved through the long process of natural evolution, containing a unique combination of four distinctive parts, namely, sepals, petals, stamens, and pistils, in a certain order (concentric whorls) that determine its morphology. Besides that, the number, shape, and size of these floral organs also determine the flower morphology. The arrangement and integration of these floral organs might be required for proper functions of the flower such as fertility, which leads to the yield of food crops. The unique flower morphology and development can be explained on the basis of the ABC model [35]. The unique flower morphology and development are regulated by ABC genes that encode the respective proteins in charge of the involved function. The ABC gene function is provided by MADS-box genes such as *AP1*, *AP2*, *AP3*, *PI*, and *AG*. This is supported by other studies that indicated that MADS-box genes are involved in the regulation of ABC genes, leading to changes in flower morphology [36,37,38,39]. Alejandra et al. found the *AP1* gene involved in sepal and petal formation and determined its development [24]. Heterologous expression of two marigold AP1 (*TeAP1-1* and *TeAP1-2*) genes in *Arabidopsis* showed their expression mainly in floral organs, which resulted in early flowering [40]. Similar phenomena were also found in transgenic tobacco on overexpressing two tobacco *AP1* promoters (*NtAP1La* and *NtAP1Lb1*) [41]. In this study, we found that expression of the class A homeotic gene *NtAP1* was upregulated in 35S::MdTFL1 transgenic tobacco. Thus, the *AP1* and *AP2* genes together provided the A function required to specify sepals [42].

Finally, we observed that transgenic lines set more fruits and seeds than WT lines with no difference in shape and size. On the basis of the fact that transgenic lines set a higher number of inflorescence and flowers, transgenic lines produced more branches than WT. Moreover, the transgenic lines set more seeds. This may be because transgenic lines own a new build-up in the flower structure suitable for pollination and fertilization as described above (Section 2.5). The exhibition of the ‘down-pin’ type of filament-style position in the flowers of transgenic lines might be suitable for pollination because the stigma was located at a lower position than the anther, thus helping the anther to meet the stigma easily when it naturally falls down during pollination. The heterologous production of a ginsenoside saponin resulted in stunted growth, and a failed set seed was reported in transgenic tobacco. In support of this, the authors indicated that the failed set seeds might be a result of strong autotoxicity of ginsenoside saponin compounds that were heterologously produced in transgenic tobacco [43]. Taken together, this finding reinforces our understanding of the interaction of genotype–phenotype and indicates the importance of genetic effects on morphology.

## 4. Materials and Methods

### 4.1. Plant Materials and Growth Conditions

The seeds of *Nicotiana tabacum* were surface-sterilized with 70% ethanol for 2 min, then with 2.0% sodium hypochlorite (NaOCl) solution containing 0.01% Tween20 (Sigma-Aldrich, Munich, Germany) for 10 min, and finally rewashed several times with sterile water. The sterilized seeds were germinated on Petri dishes with half-strength Murashige and Skoog (MS) medium basal salt mixture (pH 5.7; Duchefa, Haarlem, the Netherlands), 1% (*w*/*v*) sucrose, and 0.4% (*w*/*v*) gelrite. Plates were then kept in the dark at 4 °C for 3 days for stratification. They were then placed in a culture room at 25 °C with 16 h/8 h (light/dark) photoperiod with light intensity of 100 μmol m^−2^ s^−1^ for 2 weeks. Aseptic seedlings of *N. tabacum* were transferred into a 450 mL flask containing ½ MS at 25 °C for another 4 weeks.

### 4.2. Construction of Overexpression Vectors

Total RNA was isolated from various tissues of ‘Fuji’ apple cultivars using the CTAB method [44]. First-strand cDNA was synthesized via reverse-transcription PCR (RT-PCR) according to the manufacturer’s instructions (PrimeScript™ 1st strand cDNA Synthesis Kit, Cat. #6110A, Takara, Kusatsu, Japan) using specific primers (Table S1). For cloning of genes into an expression vector, specific primers were designed for PCR amplification to contain KpnI and BamHI restriction enzyme sites at the 5′ end of forward and reverse primers, respectively. The PCR products were analyzed on 0.8% agarose gels, and the target band of these genes was recovered from gels and purified using a fragmented DNA purification kit (iNtRON MEGAquick-spin™ Plus, Seongnam, Korea). The fragment was then ligated into the pGEM^®^-T Easy vector (Cat. # A1360, Promega, Madison, WI, USA) and transformed into *Escherichia coli* DH5α competent cells (Cat. #9057, Takara, Kusatsu, Japan), and the plasmid DNA was then sequenced (Macrogen, Seoul, Korea). The sequence information of *MdTFL1* (GenBank Accession No. AB052994.1) was obtained from the NCBI database. *MdTFL1* was digested with BamHI and KpnI restriction enzymes, and they were then cloned into the binary vector pCAMBIA1300 (CAMBIA, Canberra, ACT, Australia), which was digested with the same restriction enzyme. The *MdTFL1* gene was ligated into the binary vector in an antisense-oriented manner or revert direction (antisense) to produce the recombinant expression vector 35S::MdTFL1. The resulting construct was introduced into *Agrobacterium tumefaciens* EHA105 using the freeze–thaw method. After transformation, *A. tumefaciens* EHA105 was spread on the LB agar medium containing 50 μg/mL kanamycin and 100 μg/mL rifampicin, and it was cultured in the dark at 28 °C for 2 days. The transformed *A. tumefaciens* EHA105 colonies were picked, inoculated in 5 mL liquid LB medium containing antibiotics, and grown at 28 °C with shaking at 200 rpm in the dark overnight. The plasmid DNA of transformed *A. tumefaciens* EHA105 was isolated and analyzed for the presence of target genes in the expression vector via enzymatic digestion (Figure S2).

### 4.3. Tobacco Transformation and Establishment of Transgenic Plant

Leaf disks of in vitro tobacco plants were transformed with 35S::MdTFL1 using an *Agrobacterium*-mediated transformation method [45]. Briefly, leaf disks were cut to a size of 0.5 cm^2^ and they were soaked with the transformed *Agrobacterium* in a liquid co-cultivation medium MSCO (4.3 g/L MS medium including B5 vitamins, 30 g/L sucrose, 1 mg/L BAP, 0.1 mg/L NAA) plus 100 µM acetosyringone. Following *Agrobacterium*-mediated transformation, leaf disks were placed upside-down (adaxial contacted to medium) on solid MSCO medium containing 4 g/L gelrite in the dark for 3 days. The explants were washed with sterile distilled water 3–4 times to remove excess *Agrobacterium*, and the final washing was performed with sterile distilled water + 500 mg/L cefotaxime for 10 min with gentle shaking. The explants were blotted onto sterile filter 3M paper before transferring to the selection medium MSSE (MSCO + 250 mg/L cefotaxime, 100 mg/L hygromycin B), and they were cultured at 25 °C for 2~3 weeks in the dark for callus induction until adventitious shoots appeared and were then exposed to light for 1 more week. The putative transgenic shoots were cut at the base from the callus, transferred to MSSE, and cultured with a 16 h/8 h (light/dark) photoperiod and light intensity 100 μmol∙m^−2^∙s^−1^ subculture every 3 weeks. For obtaining whole plantlets, the shoots were transferred to the rooting medium MSR (4.3 g/L MS medium including B5 vitamins, 30 g/L sucrose, 0.1 mg/L IBA, 250 mg/L cefotaxime, 100 mg/L hygromycin B, and 4 g/L gelrite). The leaves of putative seedlings were cut off for genomic DNA extraction and PCR. The homozygous transgenic lines carrying 35S::MdTFL1 were transplanted to the soil for phenotypic and gene expression analysis. Plants were grown in a glasshouse at 25 °C under long-day conditions.

### 4.4. Genomic DNA Extraction and Genomic DNA PCR

The genomic DNA of tobacco leaves was extracted using the DNeasy^®^ Plant Mini Kit (Cat. #69204, Qiagen, Hilden, Germany) according to manufacturer’s instructions. The concentration and quality of genomic DNA were detected via UV spectrophotometry and electrophoresis on a 0.8% agarose gel, which was visualized by ethidium bromide staining. Genomic DNA PCR was performed using Maxime^™^ PCR PreMix (i-StarTaq) (Cat. #25167, Intron, Seongnam, Korea). The PCR products were visualized via electrophoresis on a 1.0% agarose gel.

### 4.5. RNA Extraction and Quantification of Gene Expression

Total RNA was isolated from tobacco leaves using an RNA Plant Mini Kit (Cat. #74904, Qiagen, Germany). The RNA samples were treated with DNase (TURBO DNA-free™ Kit, Cat. #AM1907, Invitrogen, Carlsbad, CA, USA). The first-strand cDNA was synthesized from 1.0 μg of total RNA using a PrimeScript™ 1st Strand cDNA Synthesis Kit with oligo dT primer according to the manufacturer’s instructions (Cat. #6110A, Takara, Japan). qPCR was conducted as previously described [46]. The relative expression levels of target genes were normalized to an *NtActin* gene (GenBank Accession No. U60495.1) as a reference gene. The primer sequences for qPCR reactions are listed in Table S2.

### 4.6. Measurement of Leaf Area and Leaf Mass per Area (LMA)

The leaf area of tobacco plants was measured with an LI-3100 area meter (LI-COR Inc., Lincoln, NE, USA). For calculation of LMA, the leaves were punched using a 1.6 cm diameter-cork border. The leaves were punched-off with 6 holes per leaf with no vein, and the punched-off leaves were weighed. LMA was calculated using the following formula: $\mathrm{LMA}=\frac{\mathrm{LM}}{A}$, where LM is the leaf mass of punched-leaf (mg) and A is the area of punched-leaf (cm^2^) according to the following mathematical function: $A={\pi r}^{2}$ (r = ½, d = 0.8 cm; π = 3.14).

### 4.7. Light Microscopy

The procedures were modified from a previously reported method [47]. Tissues were fixed in 2.5% glutaraldehyde (*v*/*v* in a 0.1 M phosphate buffer) at pH 7.2 in the presence of 4% sucrose (*w*/*v*) for 24 h. After three rinses (30 min, each) with the above buffer, the tissues were dehydrated in gradient concentrations of alcohol, transferred to propylene oxide, and embedded in historesin. Semi-thin sections (2.5 µm) prepared using an ultra-microtome were collected on glass slides, and the periodic acid–Schiff (PAS) polysaccharide-specific reaction was performed. A PAS-positive reaction shows a red color. Sections for staining were first plunged in 1% periodic acid (*w*/*v*) for 30 min, in Schiff’s reagent for 40 min, and finally in 5% sodium bisulfite (*w*/*v*) for 35 min. The sections were then rinsed in distilled water, dried on a warm plate, and mounted in Histomount. Negative control was performed by omitting the oxidation step with periodic acid, and it was observed with a light microscope (Axioskop 2, Carl Zeiss, Oberkochen, Germany).

### 4.8. Statistical Analysis

The results are expressed as mean  ±  standard deviation. Significant differences between multiple groups were determined using Tukey’s test. *p*  <  0.05 indicates significant differences.

## 5. Conclusions

Antisense expression of apple *MdTFL1* gene in tobacco promoted early flowering by repressing its function as a floral repressor, which resulted in reducing the duration of vegetative phase in *Arabidopsis*, suggesting that its function as a floral promoter was conserved in plants. *MdTFL1* is known to control the expression level of genes involved in controlling flowering, leading to disturbing the balance of endogenous *FT* paralogs, including floral inducers and inhibitors. Our results showed that the antisense expression of *MdTFL1* induced upregulation of multiple flower meristem identity genes, such as *NtSOC1*, *NFL*, *NtAP1,* and *NtFTs*, and downregulation of vegetative axillary meristems *NtCEN* and *NtCCDs* mediating the biosynthesis of Strigolactones (SLs). Another key finding was the distinctive phenotypic changes of overall plant architecture observed in lateral shoot outgrowth, influencing leaf and flower morphology and reproductive organs in transgenic plants. These results indicated that when modern genetic engineering was applied for breeding, besides achieving the already-known desired traits, there might be traits unknown that might appear. Thus, it is necessary to consider based on the well-known knowledge indicated by previous studies, and further studies should also be conducted. Our findings further extend the knowledge of shortened breeding, and we propose that these could be used in approaches for the improvement of plant architecture.

## Data Availability

Not applicable.

## Supplementary Materials

The following supporting information can be downloaded at https://www.mdpi.com/article/10.3390/ijms23116006/s1.
