# Supplementary material for: Antisense Expression of Apple TFL1-like Gene (MdTFL1) Promotes Early Flowering and Causes Phenotypic Changes in Tobacco
Source: Int J Mol Sci. 2022 May 26;23(11):6006. doi: 10.3390/ijms23116006 (PMC9181507; doi:10.3390/ijms23116006)
Supplement: Supplementary file 1 [file ijms-23-06006-s001.zip › ijms-1727471-supplementary.pdf]

Supplementary Materials

Table S1. Sequences of primers used for gene cloning and genomic DNA PCR

Table S2. Sequences of primers used for qRT-PCR in *Nicotiana tabacum*

Table S3. Leaf Mass Per Area (LMA) measurements

Figure S1: DNA sequence of *MdTFL1* cloned in this study

Figure S2. Gene cloning and expression vector construction

Figure S3: Phenotype and flowering time of T1 plants

Figure S4. Antisense expression of *MdTFL1* in *Arabidopsis thaliana*

Figure S5**.** Comparison of flower tubo size in tobacco

Table S1. Sequences of primers used for gene cloning and genomic DNA PCR

Gene	Encoded protein		Primer sequence (5'-3')	GenBank
MdTFL1	TFL1-like protein	F	GGGGTACCATGAAAAGAGCCTCGGAG	AB052994.1
		R	CGGGATCCCTAGCGTCTTCTAGCTG	
hptII 563bp	hygromycin phosphotransferase gene	F	CTCGGAGGGCGAAGAATCTC	KU561939.1
		R	CAATGACCGCTGTTATGCGG	

Table S2. Sequences of primers used for qRT-PCR in *Nicotiana tabacum*

Gene	Encoded protein		Primer sequence (5'-3')	GenBank
NtAct	Actin (Tob103)	F	TGGCTCAACCATGTTCCCTG	U60495.1
		R	CCGGTGGAGCAACAACCTTA	
NtAP1	Apetala 1 protein	F	TCTCAGTGCTTTGTGACGCT	JQ686939.1
		R	GCGTCTCTCTGCGTATGAGT	
NtSOC1	MADS-box protein SOC1-like	F	GGTGGCCTTCAACAGAGACA	JQ686938.1
		R	GGGACGCCTTATTCTGCACT	
NFL1	LEAFY/FLORICAULA (LFY)	F	GCTTCTAGCTCGGTTTCTGGT	JQ686928.1
		R	AGGGGCAAGCAAAGTCAGAA	
NtFT1	Flowering locus T1	F	CAACCAACCTAGGGTTGACATT	JX679067.1
		R	GTTTGGGTTGCTTGGGGTTG	
NtFT2	Flowering locus T2	F	TTCGGCAATTGGATCGAGAGG	JX679068
		R	GGCAACAGGCAAATTGAGACC	
NtFT3	Flowering locus T3	F	GGAGCACACTTCGGGAATGA	JX679069
		R	TCGAGTCAATTGTCGAAACAGC	
NtFT4	Flowering locus T4	F	GGCGTCAGAATTTCAGCACA	JX679070
		R	GCAATTGAAGTAAACAGCAGAAACG	
NtCET2	CEN-like protein 2	F	ACGCGTAAATTCGCAGAAGA	AF145260.1
		R	TTCTGGCAGCAGTTTCCCTC	
NtCET4	CEN-like protein 4	F	GGTATGAAATGCCAAGGCCA	AF145261.1
		R	TCTTCTGAGAATTTGCGCGT	
NtCCD8	Carotenoid cleavage dioxygenase 8	F	AGTTGTAGAGGAGGACCAGCA	NM_001325897.1
		R	CTTAGTGGCATTTCCGGCAC	
NtCCD1-3	Carotenoid cleavage dioxygenase 1-3	F	CTCGATTTGGAGTCCTCCCG	KM605431.1
		R	ATCTCCCTCCTCCCAAGCAT	
NtCCD4-1	Carotenoid cleavage dioxygenase 4-1	F	GGATCCCAACAATTCGGCAG	KM605432.1
		R	GGGGACTTTGCATCCATGAC	
NtCCD4-2	Carotenoid cleavage dioxygenase 4-2	F	CAGACTCGGGGAAAATCCCT	KM605433.1
		R	ATTGATCCCGGGCACATCAA	

Table S3. Leaf Mass Per Area (LMA) measurements

Genotype	Leaf No	Area	Subtotal	Max Width (cm)	Average Width (cm)	Length (cm)	Length/Max Width Ratio
WT	1	90.2	90.2	8.1	4.6	19.5	2.41
	2	115.2	205.4	9.6	5.4	21.5	2.24
	3	97.4	302.7	8.8	5.5	17.8	2.02
	4	133.4	436.1	9.8	6.2	21.5	2.19
	5	68.5	504.6	7.3	4.4	15.4	2.11
	6	65.4	570.0	6.8	4.2	15.7	2.31
	7	94.1	664.1	8.2	5.2	18.2	2.22
Average		94.9	396.2	8.4	5.1	18.5	2.21
35S::MdTFL1	1	52.3	52.3	5.9	3.4	15.4	2.61
	2	53.0	105.3	5.6	3.3	16.2	2.89
	3	69.4	174.7	6.5	3.9	17.9	2.75
	4	59.8	234.5	5.9	4.0	15.0	2.54
	5	89.3	323.7	7.3	4.5	19.8	2.71
	6	71.4	395.1	6.4	4.2	17.0	2.66
	7	49.1	444.1	5.9	3.4	14.5	2.46
	8	68.4	512.6	6.4	4.0	17.2	2.69
	9	38.6	551.2	4.5	2.4	16.3	3.62
	10	34.6	585.8	4.6	2.7	13.0	2.83
Average		58.6	337.9	5.9	3.6	16.2	2.78

***Malus x domestica* MdTFL1 mRNA for TFL1-like protein, complete cds**
GenBank: KT189149.1
Gene: 1..772
CDS: 40..558

```
CCTCTCTCTCTCTCTCTCTCTCTCTCTCTCTCTCTTAAAATGAAAAGAGCCTCGGAGCCTCTGGTTG
TTGGGAGAGTGATAGGAGATGTTCTTGATTCCTTCACTGCAACAACAAAAATGTCTGTCACTTACAA
CACCAAGCTAGTCTGCAATGGACTTGAGCTCTTTCCTTCTGTTGTCACAGCCAAACCTAGAGTTGAG
ATTCAAGGAGGGGATATGAGATCTTTCTTTACTTTGGTGATGACCGACCCAGATTTTCCTGGCCCTA
GTGATCCTTATCTAAGGGAGCACCTGCACTGGATTGTGACAGACATTCCAGGCACCACAGATGCCAC
ATTTGGAAGAGAGGTGGTGAGTTATGAGATGCCGAAGCCCAACATTGGCATCCACCGGTTTGTGTTT
GTTCTTTTCAAGCAGAATCAAAGACAATCAATCAACACACCTTCCTCGAGGGATCACTTCAGCACTC
GAAGCTTCGCGGCTGAAAATGACCTGGGTCTTCCTGTCGCTGCCGTCTACTTCAACGCGCAGAGAGA
AACTGCAGCTAGAAGACGCTAGCTAGTAGCTCTACCCAGAACTCCTCCATCCATTATCCATATATAT
GTTAAATAAAGGCTTCTTTAGAGATAGGCCATTGTAACTTTTGTTTCCCAATAACCTAAATTTTAAC
TTATTGACATGTGAGAAAATAAGTAACACGTTATTAATTATTTACAATGTATGCCACAATATTAATT
ATGTTAAATTAATTATTATTACCAAAAATAATTAT
```

Figure S1. **DNA sequence of *MdTFL1* cloned in this study**. CDS region starts with ATG and stops at TAG, underlined sequences were used for design-specific primers.

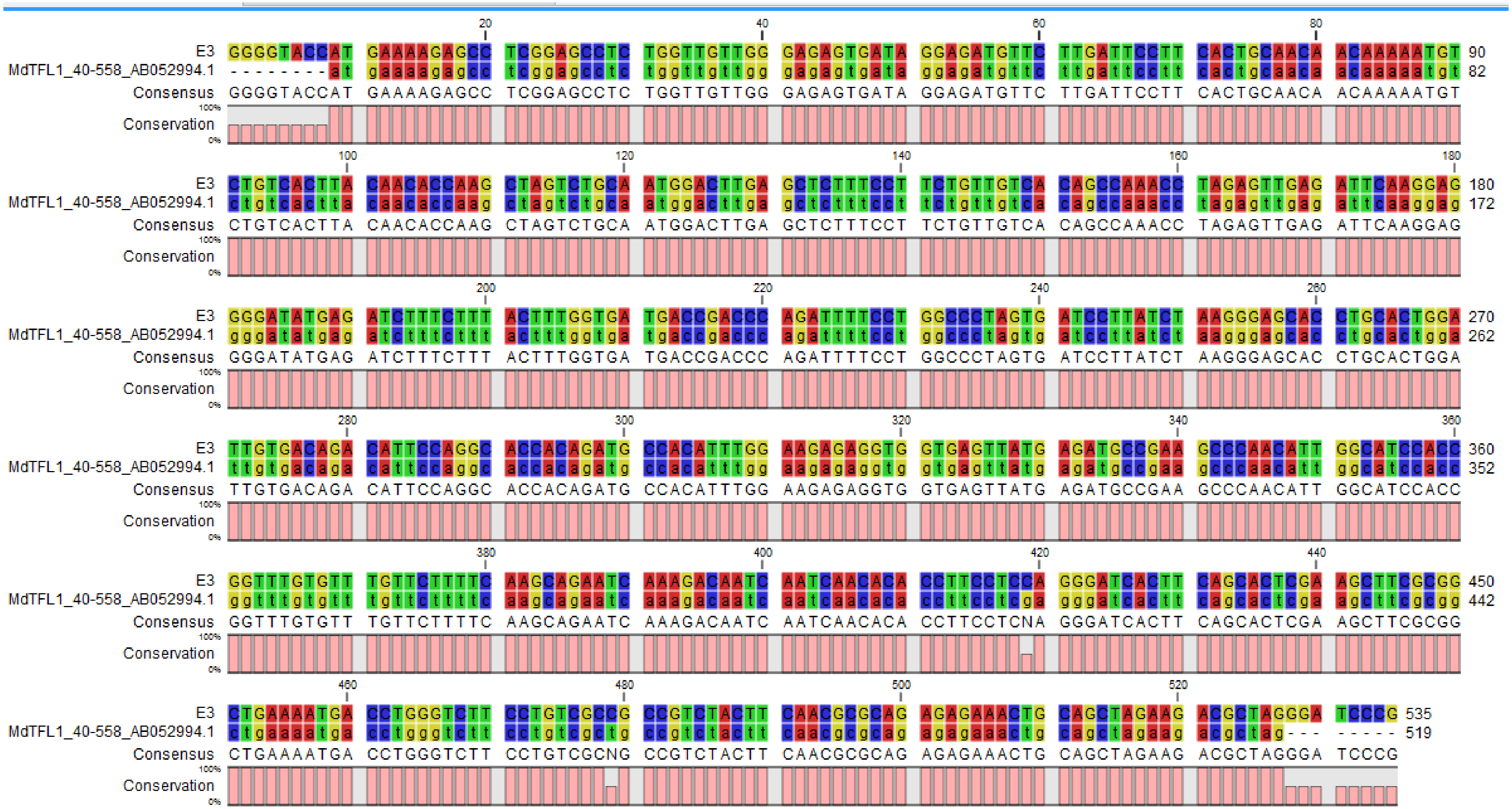

Alignment of isolated *MdTFL1* (E3) to the known sequence of original DNA (GenBank: AB052994.1)

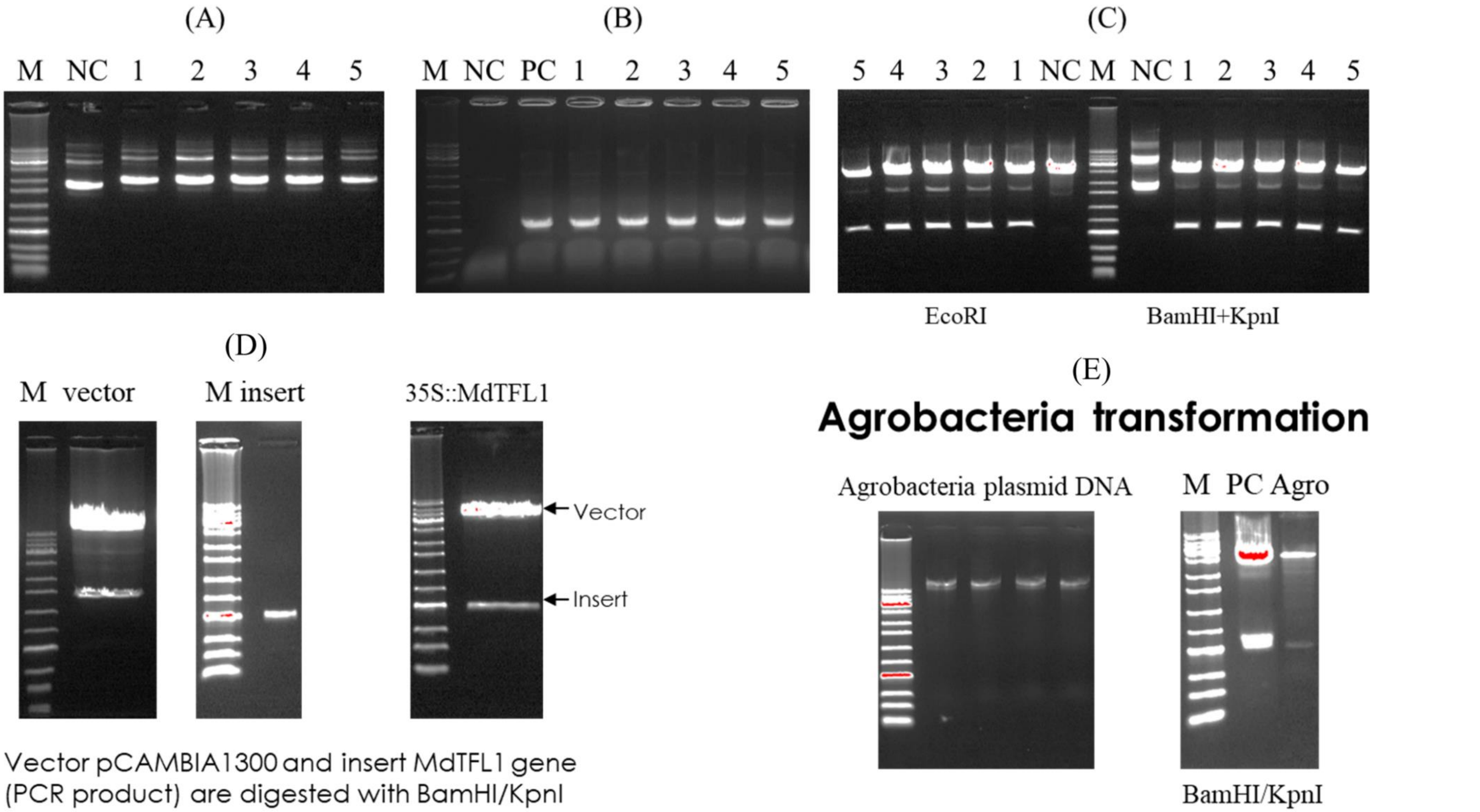

Figure S2. Gene cloning and expression vector construction. Isolated CDS of MdTFL1 containing a KpnI site at the 5'end and a BamHI site at the 3'end were used for gene cloning. (A) Plasmid DNA of *MdTFL1* in pGEM Teasy vector. (B) Colony PCR of transformed E.coli harboring MdTFL1::pGEM Teasy. (C) MdTFL1::pGEM Teasy was digested with EcoRI and BamHI+KpnI. (D) *MdTFL1* was ligated to binary vector pCAMBIA1300 resulting in 35S::MdTFL1 expression vector and confirmed by BamHI+KpnI double enzyme digestion. (E) Transformation of the expression vectors into *A. tumefaciens* EHA105. The plasmid DNA of expression vector 35S::MdTFL1 in the transformed *A. tumefaciens* EHA105 was isolated and then double-checked by restriction enzyme digestion BamHI+KpnI.

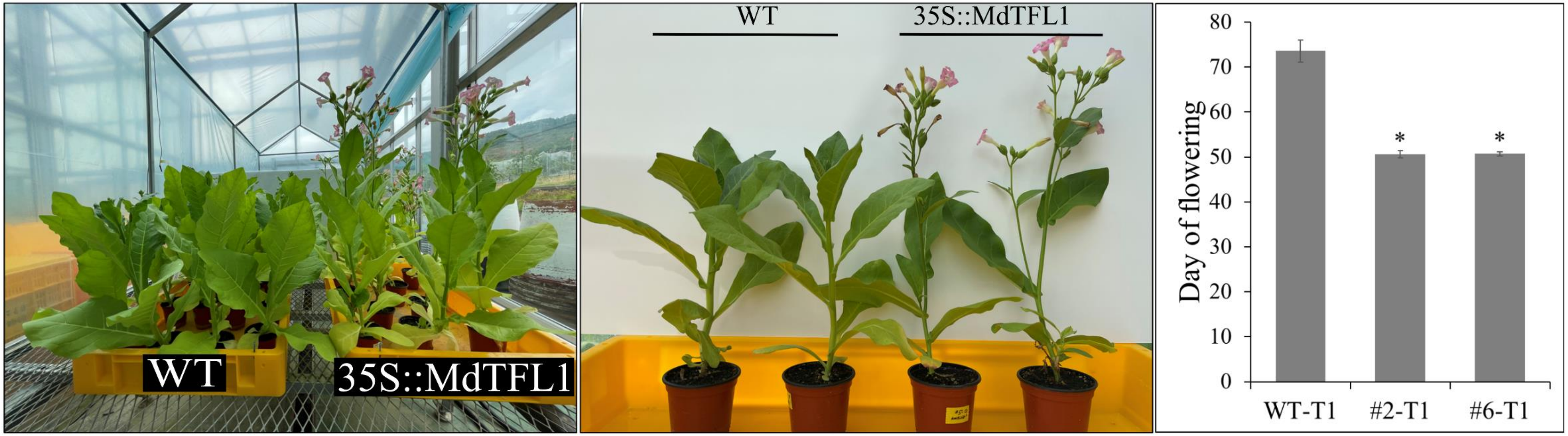

Figure S3. Phenotype and flowering time in T1 plants

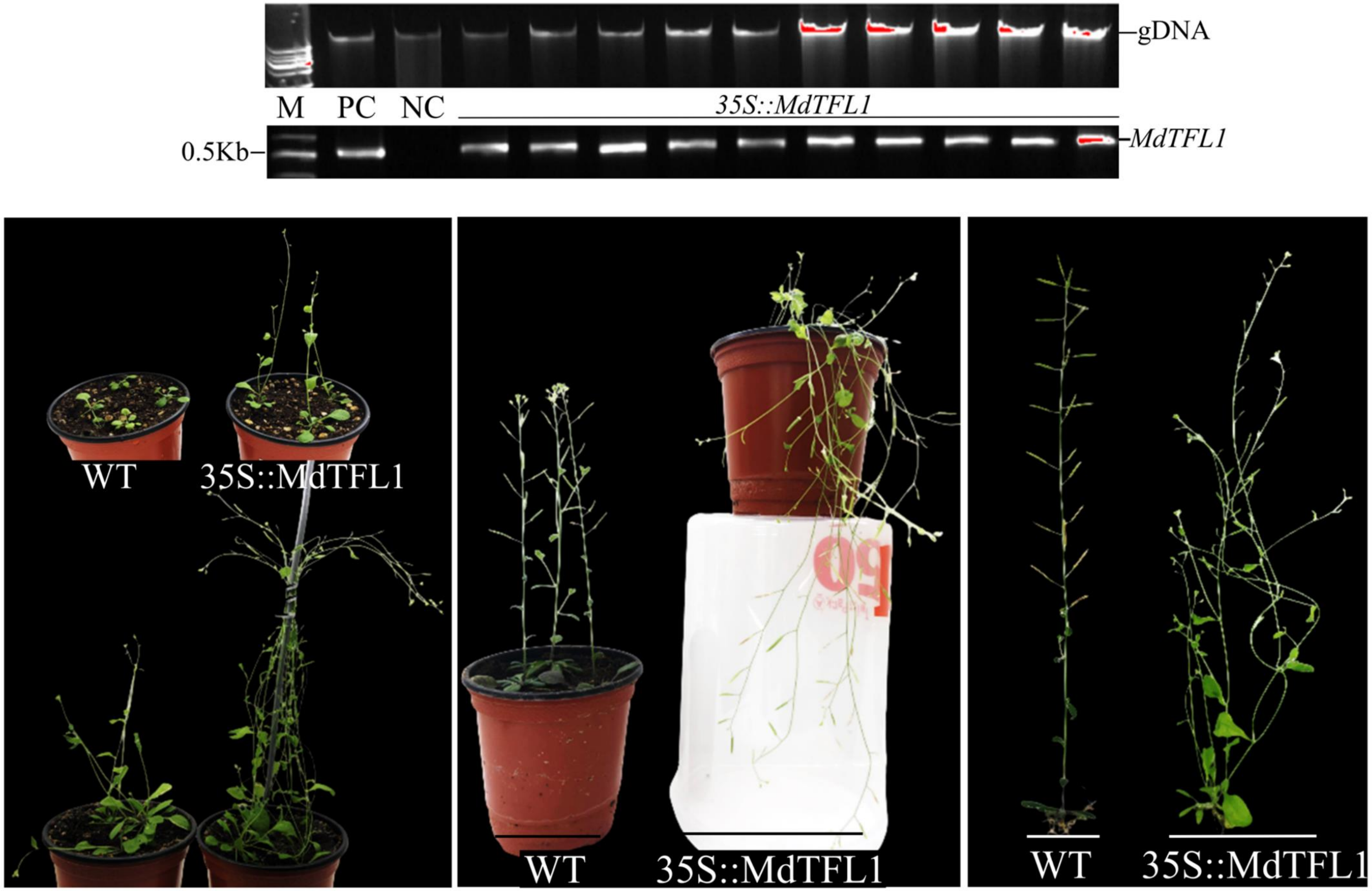

Figure S4. Antisense expression of MdTFL1 in *Arabidopsis thaliana*

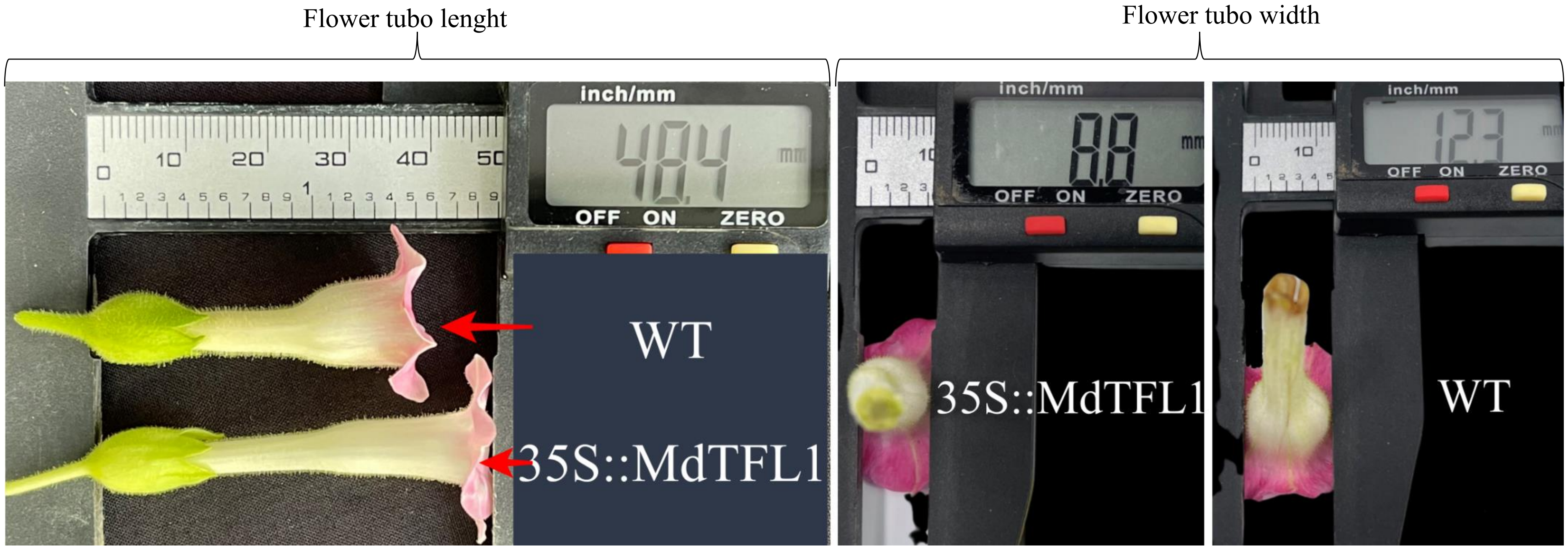

Figure S5. Comparison of flower tubo size in tobacco
